# Single Image Defogging Method Based on Image Patch Decomposition and Multi-Exposure Image Fusion

**DOI:** 10.3389/fnbot.2021.700483

**Published:** 2021-07-07

**Authors:** Qiuzhuo Liu, Yaqin Luo, Ke Li, Wenfeng Li, Yi Chai, Hao Ding, Xinghong Jiang

**Affiliations:** ^1^College of Automation, Chongqing University, Chongqing, China; ^2^National Engineering Laboratory for Highway Tunnel Construction Technology, Chongqing, China; ^3^China Merchants Chongqing Communications Technology Research & Design Institute Co., Ltd., Chongqing, China; ^4^College of Automation, Chongqing University of Posts and Telecommunications, Chongqing, China

**Keywords:** image defogging, gamma correction, multi-exposure image fusion, image patch, base and detail layers

## Abstract

Bad weather conditions (such as fog, haze) seriously affect the visual quality of images. According to the scene depth information, physical model-based methods are used to improve image visibility for further image restoration. However, the unstable acquisition of the scene depth information seriously affects the defogging performance of physical model-based methods. Additionally, most of image enhancement-based methods focus on the global adjustment of image contrast and saturation, and lack the local details for image restoration. So, this paper proposes a single image defogging method based on image patch decomposition and multi-exposure fusion. First, a single foggy image is processed by gamma correction to obtain a set of underexposed images. Then the saturation of the obtained underexposed and original images is enhanced. Next, each image in the multi-exposure image set (including the set of underexposed images and the original image) is decomposed into the base and detail layers by a guided filter. The base layers are first decomposed into image patches, and then the fusion weight maps of the image patches are constructed. For detail layers, the exposure features are first extracted from the luminance components of images, and then the extracted exposure features are evaluated by constructing gaussian functions. Finally, both base and detail layers are combined to obtain the defogged image. The proposed method is compared with the state-of-the-art methods. The comparative experimental results confirm the effectiveness of the proposed method and its superiority over the state-of-the-art methods.

## 1. Introduction

In bad weather, small floating particles (such as dust, smoke, etc.) in the air seriously degrade image quality. The color and details of scene are blurred in degraded images (Li Y. et al., 2017), affecting the performance of the applications closely related to image quality, such as outdoor video monitoring, remote sensing, and so on. Therefore, image defogging has become an important application of computer vision.

**Figure 1:**
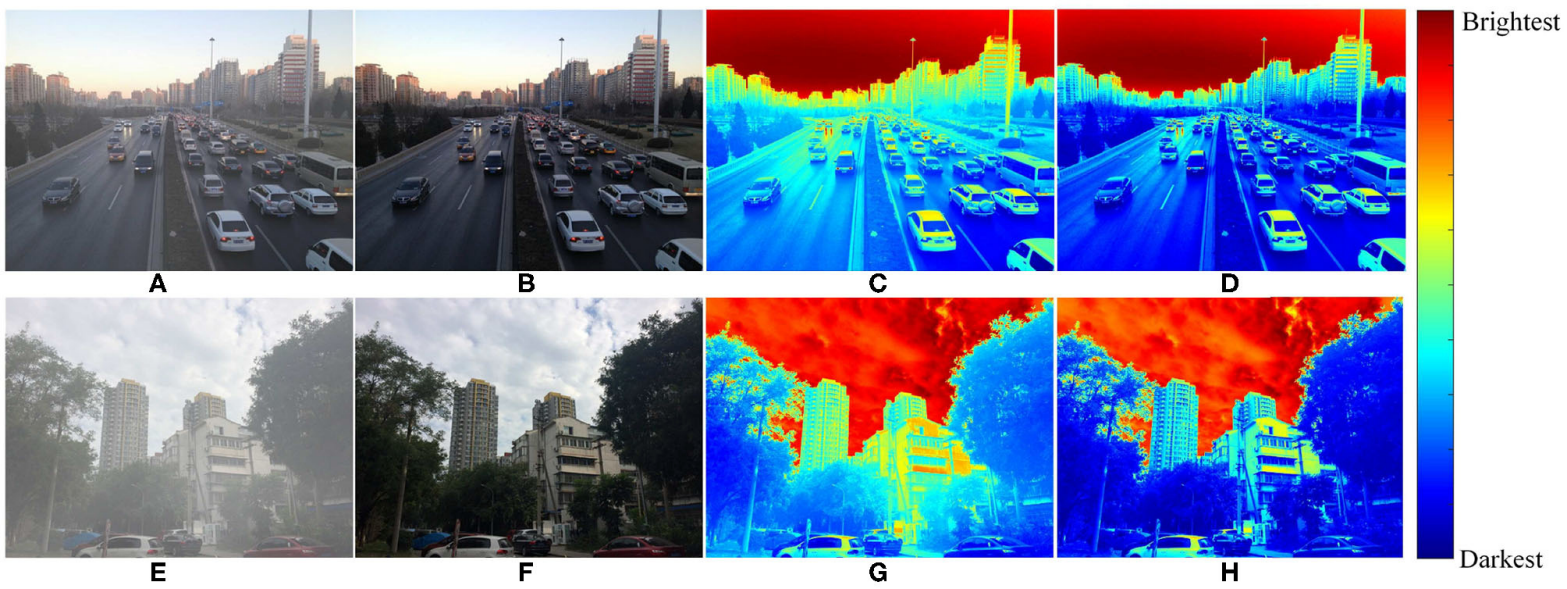
Foggy and fog-free images and their heat maps. **(C,G)** Represent the heat maps of foggy images **(A,E)**, respectively. **(D,H)** Represent the heat maps of fog-free images **(B,F)**, respectively.

As a branch of image processing techniques, image defogging techniques can effectively reduce the adverse effects of fog/haze to enhance image contrast and visibility. As shown in Figures 1A,E represent two foggy images, and Figures 1B,F represent the corresponding fog-free images of Figures 1A,E. The heat maps of both foggy and fog-free images are shown in Figures 1C–H, respectively. The overall brightness of foggy images Figures 1C,G is higher than the corresponding brightness of fog-free images Figures 1D,H. Compared with fog-free images, the feature information of foggy images is obviously blurrier, so it is necessary to remove fog/haze for the effective restoration of the captured feature information (Mehrubeoglu et al., 2016). There are many existing image defogging methods, which can be categorized into image enhancement-based, image restoration-based, and image defogging based on deep learning methods.

Most of image restoration-based defogging methods rely on the responses of atmospheric degradation models. These methods need to extract the a priori information of foggy images. Based on the dark channel prior (DCP) method, the a priori law of dark primary color is first obtained by analyzing a large number of haze-free outdoor images, and then the corresponding fog density is estimated (He et al., 2009). Based on single image defogging methods, variable surface shading is added to an atmospheric scattering model. This method assumes that the surface shading and transfer function are statistically independent. According to this assumption, an atmospheric scattering model is analyzed. So, the transfer function is obtained and haze/fog is removed from foggy images (Fattal, 2008). The contrast of input images is enhanced to improve the image visibility (Tan, 2008). In addition, fast image restoration method (Tarel and Hautiere, 2009) and Bayesian defogging method (Nishino et al., 2012; Ju et al., 2019) were proposed. Fog density changes with the depth of scene, so the degradation of image quality also changes in space. Physical degradation models need the corresponding a priori knowledge to obtain the scene depth information. Scene depth information is not only used to estimate the fog/haze distribution, but also affects the defogging performance. The a priori knowledge of physical degradation models can not be directly applied to any scene, so the acquisition of scene depth information is unstable. Without relying on the scene depth information, image enhancement-based defogging methods can effectively achieve image defogging.

With the development of deep learning, deep learning has been applied to image defogging. Image defogging methods based on deep learning are divided into non end-to-end and end-to-end. Non end-to-end methods used convolutional neural network (CNN) to estimate parameters in an atmospheric scattering model and taken parameters as the output. Parameters are introduced into the atmospheric scattering model for image restoration (Cai et al., 2016). End-to-end defogging methods input a foggy image into CNN and the defogged image directly output (Li B. et al., 2017).

Image enhancement-based defogging methods regard image degradation as the lack of contrast and saturation. The detailed information in foggy scenes can be improved by image enhancement. These methods do not need to consider the physical causes (such as fog/haze) of image degradation, and can effectively avoid the a priori estimation of the scene depth and depth mapping process. Representative defogging methods include: histogram equalization (Reza, 2004; Thomas et al., 2011), retinex-based methods (Rahman et al., 2004), homomorphic filter (Yu et al., 2015), wavelet transform (Rong and Jun, 2014; Jin et al., 2018a), and image fusion-based defogging methods (Li Y. et al., 2017; Galdran, 2018). These methods enhance both image contrast and saturation, so as to improve image visual quality. The detailed image information is first extracted from a single foggy image, and then fused to restore the details of the blurred areas. However, the defogging result obtained by the simply fusion of the two images cannot preserve all the detailed information of the scene in the original foggy image. To improve the detail preservation ability of image fusion techniques in the defogging process. Galdran (2018) introduced multi-exposure fusion techniques into image defogging. Multiple images with different exposure levels were extracted from one image by gamma correction, and saturation and contrast were considered as the weights of fusion. Multi-exposure fusion method was used to improve image visual quality from the global enhancement. However, some local information may be ignored in the global enhancement process, which affects the definition of the final output images. Therefore, it is necessary to optimize both global and local exposure, respectively (Qi et al., 2020).

To solve the above issues, this paper proposes a single image defogging method based on image patch decomposition and multi-exposure fusion. Since fog density is sensitive to contrast, gamma function is used to restore the details of local information by adjusting image contrast. A single input foggy image is corrected by gamma correction, so a set of underexposed images with different contrast are obtained. Spatial linear saturation enhancement is applied to the underexposed and original images, and then a set of foggy images with contrast and saturation enhancement are obtained. To retain more detailed information, images decomposition and fusion are used to enhance the detailed information of foggy images. With the help of a guided filter, each of multi-exposure images obtained after saturation adjustment is decomposed into the base and detail layers in the spatial domain. The guided filter does not damage any structure and detailed information of the processed images. In the base layer, a fixed-size moving window is used to extract image patches, and the best-quality areas are selected from each image patch for the fusion of image patches. According to the exposure features of each input image, the value of each pixel in the detail layer is estimated in the optimal exposure mode. The weight maps of both base and detail layers are constructed for image fusion. So, the fog-free image is obtained after fusing the base and detail layers. This paper has two main contributions as follows.

1. The proposed method can effectively avoid the complex process of both scene depth a priori estimation and depth mapping. A set of underexposed images are obtained by adjusting the contrast of foggy images. Spatial linear saturation adjustment is used to improve image saturation. Local features of foggy images are optimized by image patch structure decomposition to enhance the visual quality of fog-free images.
2. The proposed method can further improve the visual quality of the obtained fog-free images. Each exposure image is decomposed into based and detail layers. In the base layer, the local exposure quality is optimized by image patch structure decomposition. In the detail layer, the global exposure quality is optimized by the exposure degree evaluation model.

The rest of this paper is organized as follows. Section 2 discusses the related work; Section 3 elaborates the proposed solution in detail; Section 4 analyzes the comparative experimental results; and Section 5 concludes this paper.

## 2. Related Work

Some researchers regard image defogging as a type of image restoration, so fog-free images can be obtained by an atmospheric light scattering model (Gonzalez et al., 2014). As a representative solution, dark channel prior (DCP) method proposed by He et al. (2009) makes at least one low-intensity pixel in a color channel of the local neighborhood around each pixel. This method learns the mapping relationship between a foggy image and the corresponding scene depth, and uses the value of the learned image transmission map to retrieve a physical model, so as to obtain the fog-free image by physical model calculation. Zhu et al. (2015) established a linear model based on the a priori information of a foggy image. According to the a priori scene depth information, an atmospheric scattering model is used to estimate transmittance and restore scene radiance, so as to effectively eliminate fog from a single image. He et al. (2016) proposed a convex optimization formula for image defogging. In the proposed foggy image model, bilinear coupled foggy images and light transmission distribution term are established to directly reconstruct the fog-free image. Fan et al. (2016) constructed a two-layer Gaussian process regression model, which established the relationship between an input image and its depth information transmission. In this method, the a priori knowledge of the local image structure is learned, and the multi-scale feature vectors of the input image are mapped to the corresponding transmitted light. The training model is used to restore the fog-free image. Wang et al. (2019) found that fuzzy regions were mainly concentrated on the luminance channel of YCrCb color space. So, the texture information lacking in the luminance channel can be recovered to enhance the visual contrast of foggy scenes. Yuan et al. (2017) introduced the gaussian mixture model (GMM). Based on haze density feature maps, an input foggy image is segmented into multiple scenes. The segmentation results can effectively identify sky areas that DCP cannot handle well. In the improved DCP model (Singh and Kumar, 2017), the atmospheric veil enhancement estimation is obtained by using the joint trilateral filter, and transmission maps are redefined to reduce the color distortion. Liu et al. (2017) proposed a ground radiation suppressed haze thickness map (GRS-HTM) based on haze thickness map (HTM) to calculate the fog distribution in the foggy image. The visible bands are affected by fog density. Fog components of each band are calculated by GRS-HTM to restore the fog-free image. Fog density changes with the depth of scene, so the degradation of image quality is also spatially variable. Atmospheric degradation model depends on the depth information of the corresponding scene, but the acquisition of scene depth information is unstable. This affects the accurate estimation of fog distribution and defogging performance. Without relying on the scene depth information, image enhancement-based defogging methods were proposed.

Image enhancement-based defogging methods mainly focus on enhancing both image contrast and saturation and highlighting image details. Yu et al. (2015) converted foggy images from RGB to HSV space. The overlapped sub-patch homomorphic filter is applied to the luminance components, and the processed image is converted back to RGB space to obtain the fog-free image. Kim et al. (2017) combined DCP, contrast constrained adaptive histogram equalization and discrete wavelet transform (CLAHE-DWT). First, the estimation of transfer function is improved in DCP. Then, image contrast and definition are improved by CLAHE-DWT, respectively. Finally, images processed by CLAHE-DWT are fused to generate the enhanced image. Galdran et al. (2015) proposed an enhanced variable image dehazing (EVID) method. This method enhances the local low pixels by adjusting the gray world hypothesis. Image colors are restored by controlling saturation, and image contrast between different channels is also improved. Image fusion is an important method used in image defogging, which can effectively improve the image contrast, detail information and so on (Jin et al., 2020; Liu et al., 2020). In the same scene, since the imaging equipment cannot focus different depth objects at the same time, so multi-focus image fusion technology is used to extract different focus areas from multiple images to synthesize a clear image (Jin et al., 2018b; Liu et al., 2019b). A fusion framework decomposes the source image into high- and low-pass subbands. The high-pass subbands are processed by a phase congruency-based fusion rule, and the low-pass subbands are processed by a local Laplacian energy-based fusion rule. The fused image is obtain by inversely transforming the processed high-pass and low-pass subbands. The fused image not only contains the enhanced detailed features, but also retains the structural information of the source image (Zhu et al., 2019). Li Y. et al. (2017) first used an adaptive color normalization method to correct color distortion images, and then enhanced the local details of both original and color corrected images. Dark channel, sharpness, and saliency features were taken as the weight maps for image fusion, and the pyramid fusion strategy was used to reconstruct images. Liu et al. (2019a) first transformed the speckle noise into additive noise by logarithmic transformation. Then, the local image blocks are matched by Gray theory, the approximate low-rank matrices grouped by the similar blocks of the reference patches is obtained. Wavelet transform is used to estimate the noise variance of the noisy image. Finally, weighted nuclear norm minimization is used to the denoised image. Gao et al. (2020) obtained a set of self-constructed images with different exposure levels by segmenting atmospheric light range. Therefore, an adaptive multi-exposure image fusion method based on scale invariant feature transform (SIFT) flow was proposed. On the basis of fusion, self-constructed images with different exposure levels are adaptively selected by using two-layer visual sense selectors. Galdran (2018) applied the multi-exposure image fusion method to image defogging. The global image exposure quality is enhanced to improve the image visual quality. This method enhances the global image features, but the enhancement of local features is uncertain, which affects the image quality. On the same basis, Zhu et al. (2021) also used gamma correction to obtain a set of images with different exposure. By analyzing the global and local exposure, the weight maps are constructed to guide the fusion process. The defogged image is obtained after saturation adjustment. Zheng et al. (2020) directly adjusted the saturation of underexposed images after gamma correction, and proposed a fusion method based on adaptive decomposition of image patches. The adaptive selection of image patch size is realized by fitting both texture entropy and image patch size. High weights are assigned to image patches with good visual quality for image fusion. Similar to this method, this paper also proposes an image patch based multi-exposure fusion method for image defogging. Image restoration is achieved through the optimization of both local and global exposure quality.

Now, deep learning is widely used in image defogging. Cai et al. (2016) first applied deep learning to image defogging and proposed DehazeNet. This paper used DehazeNet to estimate a medium transmission map in an atmospheric scattering model. A hazy image as input, and outputs its medium transmission map. Then, a haze-free image is recovered by atmospheric scattering model. And a novel nonlinear activation function is proposed, the quality of recovered haze-free image is improved by this function. Zhang and Patel (2018) proposed a new single image dehazing method, called densely connected pyramid dehazing network (DCPDN). DCPDN includes two generators, which are used to generate the transmission map and the atmospheric light, respectively. A new edge-preserving densely connected encoder-decoder structure with multi-level pyramid pooling module is designed to estimate the transmission map. Then the U-net structure is used to estimate the atmospheric light.Both the transmission map and the atmospheric light are introduced into an atmospheric scattering model to restore the fog-free image. A joint-discriminator based on generative adversarial network (GAN) framework is proposed to further incorporate the mutual structural information between the estimated transmission map and the dehazed result. This kind of defogging method using network estimation parameters still needs the help of atmospheric scattering model. Li B. et al. (2017) proposed an image dehazing model built with a CNN, called All-in-One Dehazing Network (AOD-Net). This paper dosed not estimate the transmission map and the atmospheric light separately, but directly generated clear images through light-weight CNN. Qin et al. (2020) proposed an end-to-end feature fusion attention network (FFA-Net) for single image dehazing. This paper combined channel attention and pixel attention mechanism to form a novel feature attention (FA) module. FA focused more attention on the thick haze pixels and more important channel information. And local residual learning allows the less important information to be bypassed through multiple skip connections. To giving more weight to important features, an attention-based different levels feature fusion (FFA) structure is proposed, the feature weights are adaptively learned from FA.

## 3. The Proposed Image Defogging Method

**Figure 2:**
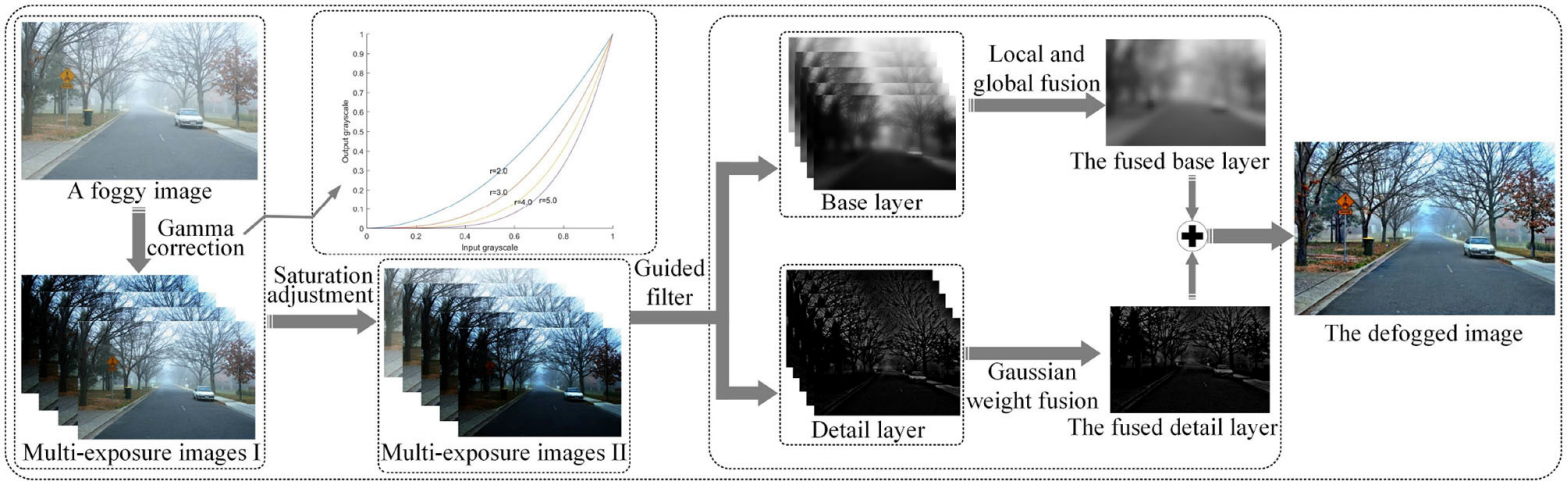
The proposed image defogging framework based on image patch and multi-exposure fusion.

As shown in Figure 2, the proposed single image defogging method performs gamma correction on an input foggy image to obtain a set of underexposed images. Both the underexposed images and the original image are enhanced by spatial linear saturation. All the images are decomposed into base and detail layers by a guided filter. A fixed-size moving window is used to extract image patches from the base layer. Low-level features such as signal strength, signal structure, and mean intensity are used to improve fusion quality. Image patches are decomposed into signal strength, signal structure, and mean intensity by a structure decomposition method. The best-quality areas of the above three low-level features are selected for fusion. The whole luminance components of each input image are used to extract exposure features, and the extracted features are applied to optimize the global exposure quality of detail layer.

### 3.1. Image Preprocessing by Gamma Function

**Figure 3:**
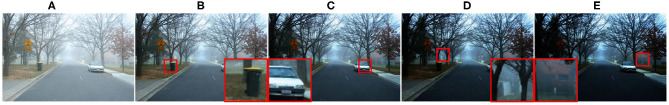
Original image is corrected by gamma function. **(A)** A foggy image, **(B)** γ = 2, **(C)** γ = 3, **(D)** γ = 4, **(E)** γ = 5.

Gamma correction is used to adjust an input foggy image *I*(*x*) nonlinearly by increasing or decreasing the exposure of the input image to change the local contrast of blurry areas.

(1) $$\begin{array}{l} I\left(x\right)\mapsto \alpha \cdot I{\left(x\right)}^{\gamma } \end{array}$$

where α and γ are positive numbers. When γ < 1, the gray level of bright areas is compressed. The gray level of dark areas is stretched to be brighter, and the whole image becomes bright, which causes the color tone of high-luminance contents to be too bright. So, the detailed contents are not obvious in human visual perception (Galdran, 2018). On the contrary, when γ > 1, the whole image darkens and a series of underexposed images are obtained, and the image details are highlighted. For the input foggy image *I*(*x*), the contrast Y of the given area Ω is shown as follows.

(2) $$\begin{array}{l} Y\left(\Omega \right)=y_{I_{\max}^{\Omega }}-y_{I_{\min}^{\Omega }} \end{array}$$

where $y_{I_{\max}^{\Omega }}=\max\left\{y_{I\left(x\right)}|x\in \Omega \right\}$ and $y_{I_{\min}^{\Omega }}=\min\left\{y_{I\left(x\right)}|x\in \Omega \right\}$. When γ > 1, a set of underexposed images are obtained by Equation (2). Gamma correction is a kind of global correction, and the contrast of some areas with moderate exposure is reduced. As shown in Figure 3, the value of γ is 2, 3, 4, or 5, respectively, four foggy images with different exposure are obtained by gamma correction. Different exposure images highlight the details of different areas.

### 3.2. Saturation Enhancement

The input foggy image *I*(*x*) is corrected by gamma ray to obtain a set of multi-exposure image sequences *Q* = {*I*_1_(*x*), *I*_2_(*x*), …, *I*_*N*_(*x*)|*N* = 5}. Each image has $I_{n}\left(x\right)=\left[I_{n}^{R}\left(x\right),I_{n}^{G}\left(x\right),I_{n}^{B}\left(x\right)\right]$. For each image, the maximum and minimum values of each pixel are calculated.

(3) $$\{\begin{array}{l} rgb_{\max}=\max\left(R,\max\left(G,B\right)\right)\\ rgb_{\min}=\min\left(R,\min\left(G,B\right)\right) \end{array}$$

When Δ = (*rgb*_max_ − *rgb*_min_)/255 > 0, the saturation P of each pixel in an image is calculated as follows.

(4) $$P=\{\begin{array}{l} \Delta /value,L<0.5\\ \Delta /\left(2-value\right),L\geq 0.5 \end{array}$$

where *value* = (*rgb*_max_ + *rgb*_min_)/255 and *L* = *value*/2. The saturation of each pixel is normalized. The same adjustment operation is performed on the three channels of RGB, and the adjustment of saturation increment for each image is within [−100, 100].

When *Increment* ≥ 0, the three channels of RGB are adjusted by Equation (5).

(5) $$\begin{array}{l} I_{n}^{\prime }\left(x\right)=I_{n}\left(x\right)+\left[I_{n}\left(x\right)-L\times 255\right]\times \alpha \end{array}$$

where α = 1/β−1 and $I_{n}^{\prime }\left(x\right)=\left[I_{n}^{R^{\prime }}\left(x\right),I_{n}^{G^{\prime }}\left(x\right),I_{n}^{B^{\prime }}\left(x\right)\right]$ represents the saturation of an image after saturation adjustment.

(6) $$\beta =\{\begin{array}{l} P,\;Increment+P\geq \text{1}\\ 1-Increment,\text{ else} \end{array}$$

When *Increment* < 0, the three channels of RGB are adjusted by Equation (7).

(7) $$\begin{array}{l} I_{n}^{\prime }\left(x\right)=I_{n}\left(x\right)+\left[I_{n}\left(x\right)-L\times 255\right]\times \left(1+\alpha \right) \end{array}$$

where α = *Increment*.

### 3.3. Multi-Exposure Image Fusion Defogging

#### 3.3.1. Image Decomposition by a Guided Filter

The input images $\left\{I_{n}^{\prime }\left(x\right)|1\leq n\leq N,N=5\right\}$ is decomposed into the base and detail layers. Luminance component *G*_*n*_ of the input image is calculated by the weighted sum of the three channels of RGB. Since a guided filter can keep edge-preservation smooth (Li et al., 2012), the base layer is obtained by a guided filter as follows.

(8) $$\begin{array}{l} B_{n}=T_{r,\delta }\left(G_{n},G_{n}\right) \end{array}$$

where *T*_*r*, δ_(*Z, H*) is a guided filter operator, *r* is the filter radius, and δ is used to control fuzzy degree. *Z* and *H* represent both input image and guide image, respectively. *G*_*n*_ represents both input image and guide image (Nejati et al., 2017). The detail layer *D*_*n*_ is obtained as follows.

(9) $$\begin{array}{l} D_{n}=I_{n}^{\prime }\left(x\right)-B_{n} \end{array}$$

#### 3.3.2. Fusion Defogging Based on Global and Local Optimization

**Figure 4:**
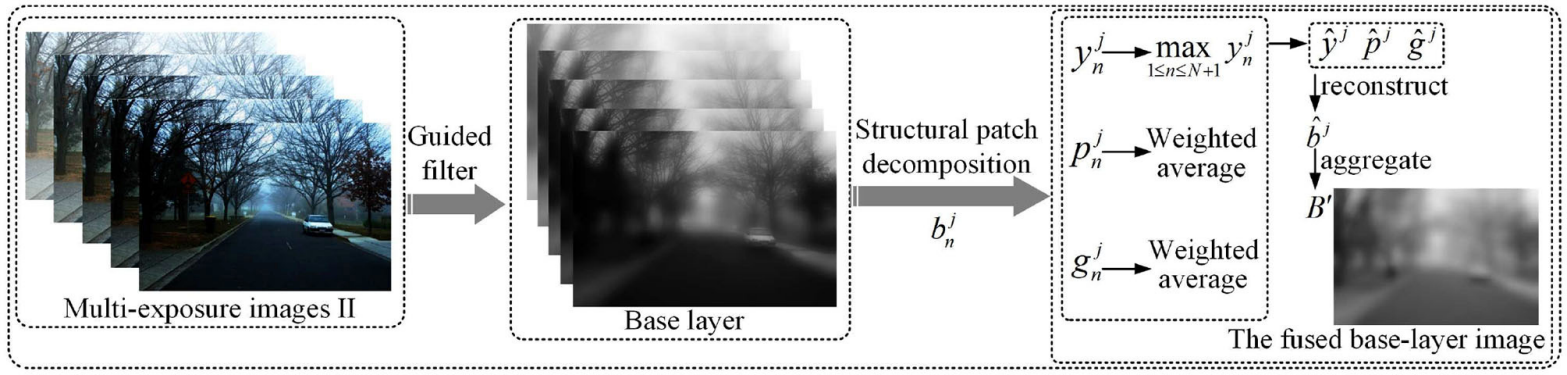
The fusion process of the base layer. $b_{n}^{j}$ represents image patches. $y_{n}^{j}$, $p_{n}^{j}$, and $g_{n}^{j}$ represent signal strength, signal structure, and mean intensity, respectively. *y*^*j*^, *p*^*j*^, and *g*^*j*^ represent the desired signal strength, signal structure, and mean intensity, respectively. ${\hat{b}}^{j}$ represents the fusion of image patches, *B*′ is the fused base-layer image.

As shown in Figure 4, the optimization of both global and local exposure is realized by structure decomposition. A fixed-size moving window is used to extract image patches $b_{n}^{j}=\left\{b_{n}^{j}|1\leq n\leq N,1\leq j\leq J\right\}$ from the base layer, $b_{n}^{j}$ represents the j-th image patch of the n-th image. Structure decomposition proposed in Ma et al. (2017) is used to decompose image patches. Image patches are decomposed into three parts by Equation (10): signal strength $y_{n}^{j}$, signal structure $p_{n}^{j}$, and mean intensity $g_{n}^{j}$.

(10) $$\begin{array}{l} b_{n}^{j}=\left\|b_{n}^{j}-\mu_{b_{n}^{j}}\right\|\cdot \frac{b_{n}^{j}-\mu_{b_{n}^{j}}}{\left\|b_{n}^{j}-\mu_{b_{n}^{j}}\right\|}+\mu_{b_{n}^{j}}\\ \text{=}\left\|{\tilde{b}}_{n}^{j}\right\|\cdot \frac{{\tilde{b}}_{n}^{j}}{\left\|{\tilde{b}}_{n}^{j}\right\|}+\mu_{b_{n}^{j}}\\ \text{=}y_{n}^{j}\cdot p_{n}^{j}+g_{n}^{j} \end{array}$$

where $\mu_{b_{n}^{j}}$ is the mean value of each image patch, and ||·|| is the *l*_2_-norm of the vector.

The highest signal strength of all image patches at the same spatial position in the image set is taken as the expected signal strength ŷ^*j*^ of the fused image patch.

(11) $${\hat{y}}^{j}=\underset{1\leq n\leq N}{\max}y_{n}^{j}=\underset{1\leq n\leq N}{\max}\left\|{\tilde{b}}_{n}^{j}\right\|$$

To obtain the expected image patch signal structure, the weighted average of the signal strength of input image patch set is calculated as follows.

(12) $$\begin{array}{l} {\hat{p}}^{j}=\frac{\sum_{n=1}^{N}P\left({\tilde{b}}_{n}^{j}\right)p_{n}^{j}/\sum_{n=1}^{N}P\left({\tilde{b}}_{n}^{j}\right)}{\left\|\sum_{n=1}^{N}P\left({\tilde{b}}_{n}^{j}\right)p_{n}^{j}/\sum_{n=1}^{N}P\left({\tilde{b}}_{n}^{j}\right)\right\|} \end{array}$$

where the weight function $P\left({\tilde{b}}_{n}^{j}\right)=\|{\tilde{b}}_{n}^{j}{\|}^{t}$ determines the contribution of each image patch to the fused image patch, and *t* ≥ 0 is an exponential parameter. When the value of *t* gets larger, the image patch with higher intensity is highlighted.

The exposure quality of each image patch in the input image is measured by a two-dimensional gaussian function.

(13) $$\begin{array}{l} G\left(\mu_{n},g_{n}^{j}\right)=\exp\left(-\frac{{\left(\mu_{n}-0.5\right)}^{2}}{2\delta_{\mu }^{2}}-\frac{{\left(g_{n}^{j}-0.5\right)}^{2}}{2\delta_{g}^{2}}\right) \end{array}$$

where δ_μ_ and δ_*g*_ are the gaussian standard deviations of the constructed two-dimensional gaussian function. δ_μ_ and δ_*g*_ control the expansion of contour along μ_*n*_ and size $g_{n}^{j}$, respectively. The expected mean intensity ĝ^*j*^ of the image patch is shown as follows.

(14) $$\begin{array}{l} {\hat{g}}^{j}=\frac{\sum_{n=1}^{N}G\left(\mu_{n},g_{n}^{j}\right)g_{n}^{j}}{\sum_{n=1}^{N}G\left(\mu_{n},g_{n}^{j}\right)} \end{array}$$

ŷ^*j*^, ${\hat{p}}^{j}$, and ĝ^*j*^ form a new vector. The fused image patch ${\hat{b}}^{j}$ is represented as follows.

(15) $$\begin{array}{l} {\hat{b}}^{j}={ŷ}^{j}\cdot {\hat{p}}^{j}+{ĝ}^{j} \end{array}$$

To optimize the local-exposure quality, a fixed-size moving window is used to extract image patches at the same spatial position from the base layer of the input image. The pixels in the overlapped image patches are averaged. The above steps of the decomposition and fusion of image patches are repeated, and then $\overset{J}{\underset{j=1}{\sum }}{\hat{b}}^{j}$ is used to obtain the fused image *B*′ of the base layer.

Two dimensional gaussian function is used to evaluate the exposure quality of *B*′ and optimize the global exposure quality of *B*′. The mixed weight E_*n,B*_ of each pixel (*x, y*) in $B_{n}^{\prime }$ is calculated as follows.

(16) $$\begin{array}{l} {\text{E}}_{n,B}\left(x,y\right)=\exp\left(-\frac{{\left(B^{\prime }\left(x,y\right)-0.5\right)}^{2}}{2\delta_{\mu }^{2}}-\frac{{\left(Ḡ-0.5\right)}^{2}}{2\delta_{g}^{2}}\right) \end{array}$$

$\hat{B}$ represents the weighted sum of each base-layer image in the input image set and its corresponding weight E_*n,B*_ in the fused image.

(17) $$\begin{array}{l} \hat{B}=\overset{N}{\underset{n=1}{\sum }}{\text{E}}_{n,B}B^{\prime } \end{array}$$

#### 3.3.3. Exposure Fusion Image Based on Gaussian Weight Method

Each luminance component is convoluted with a 7 × 7 average filter to simply calculate the exposure features φ_*n*_(*x, y*) of each pixel in multi-exposure image set, and φ_*n*_(*x, y*) is the mean intensity of a small area around the pixel (x, y). The value of each pixel in the detail layer in the optimal exposure mode is estimated by analyzing the shading changes of different pixels. The weight E_*n,D*_(*x, y*) of each pixel (x, y) in the detail layer of the n-th input image is calculated by using the exposure degree evaluation model.

(18) $$\begin{array}{l} {\text{E}}_{n,D}\left(x,y\right)=\exp\left(-\frac{{\left(\varphi_{n}\left(x,y\right)-\varphi_{0}\right)}^{2}}{2\delta_{d}^{2}}\right) \end{array}$$

where φ_*n*_(·) is the exposure feature, δ_*d*_ is the gaussian standard deviation, and φ_0_ as the best exposure constant equals the middle value of the intensity range.

The defogged image is defined as follows.

(19) $$\begin{array}{l} J\left(x\right)=\hat{B}+\omega \overset{N}{\underset{n=1}{\sum }}{\text{E}}_{n,D}D_{n} \end{array}$$

where ω ≥ 1 controls the detail intensity and local contrast of the defogged image *J*(*x*). According to the experimental results of the fusion performance, the value of ω is set to 1.1.

#### 3.3.4. Verification of Image Intensity Reduction After Defogging

Koshmieder proposed an atmospheric scattering model to solve the image degradation issues caused by fog (Gonzalez et al., 2014).

(20) $$\begin{array}{l} I\left(x\right)=t\left(x\right)J\left(x\right)+A\left(1-t\left(x\right)\right) \end{array}$$

where *I*(*x*) represents a foggy image. *J*(*x*) represents the corresponding fog-free image of *I*(*x*). A represents the global atmospheric light. *t*(*x*) is the transmitted light. *t*(*x*)*J*(*x*) describes the radiation and attenuation of the scene in the medium. *A*[1 − *t*(*x*)] is the atmospheric light formula.

Equation (20) that reduces image intensity is used to formalize foggy images. In this paper, underexposure or overexposure processing is applied to foggy images, and the corresponding exposure results are fused to obtain the image areas with good exposure quality. To meet the requirement of image intensity reduction, the proposed method is only applied to the underexposed images to reduce global exposure. When γ > 1, it is easy to verify that the fused image obtained by using $B_{n}^{\prime }=\overset{J}{\underset{j=1}{\sum }}{\hat{b}}_{n}^{j}$ always meets the requirement of image intensity reduction.

Proof:

In Zheng et al. (2020), it simply verifies that the fusion of the images obtained after gamma correction, saturation linear adjustment and image structure decomposition meets the requirement of intensity reduction *J*(*x*) ≤ *I*(*x*). The proof is shown as follows.

Given a set of gamma parameters Γ = {γ^1^, γ^2^, …, γ^*K*^|γ^*k*^ > 1}, a set of underexposed images *Q* = {*I*_1_(*x*), *I*_2_(*x*), …., *I*_*N*−1_(*x*)} is obtained. Since *I*(*x*) ∈ [0, 1], *I*(*x*)^γ^^*k*^ < *I*(*x*) is available for all pixels. Due to the invariance principle of brightness in the linear adjustment of saturation, the pixel intensity component is $I\left(x\right)=\frac{1}{3}\left(R+G+B\right)$ (Gonzalez and Woods, 1977). Therefore, for any foggy image, $I\left(x\right)=Q_{n}^{\prime }\left(x\right)$ is satisfied before and after saturation adjustment. Therefore, all the pixels after saturation adjustment satisfy ${\left(Q_{n}{\left(x\right)}^{\gamma^{k}}\right)}^{\prime }<I\left(x\right)$. Since an image patch $b_{n}^{j}\in I\left(x\right)$, all $b_{n}^{j}{\left(x\right)}^{\gamma^{k}}\in {\left(Q_{n}{\left(x\right)}^{\gamma^{k}}\right)}^{\prime }$ satisfy $b_{n}^{j}{\left(x\right)}^{\gamma^{k}}<b_{n}^{j}\left(x\right)$. Therefore, image patches can meet the requirements of image intensity reduction after gamma correction and saturation adjustment.

According to the above proof $b_{n}^{j}\in I\left(x\right)$ is satisfied for any image patch. The structure decomposition of image patches is performed on both sides of Equation (21) (Ma et al., 2017).

(21) $$\begin{array}{l} \left({\left(y_{n}^{j}\right)}^{\gamma^{k}}\cdot {\left(p_{n}^{j}\right)}^{\gamma^{k}}+{\left(g_{n}^{j}\right)}^{\gamma^{k}}\right)<\left(y_{n}^{j}\cdot p_{n}^{j}+g_{n}^{j}\right) \end{array}$$

Since $y_{n}^{j}$, $p_{n}^{j}$, and $g_{n}^{j}$ of each image patch are unit length vectors, and the initial foggy image *I*(*x*) is the input of image fusion. Therefore, the expected contrast of the fused image patch satisfies ${ŷ}^{j}=\underset{1\leq n\leq N+1}{\max}y_{n}^{j}\leq y^{j}$. Similarly, since the weight of the mean luminance is $\sum_{n=1}^{N}\left(\frac{G\left(\mu_{n},g_{n}^{j}\right)}{\sum_{n=1}^{N}G\left(\mu_{n},g_{n}^{j}\right)}\right)=1$, the expected average brightness is satisfied as follows.

(22) $$\begin{array}{l} {ĝ}^{j}=\frac{\sum_{n=1}^{N}G\left(\mu_{n},g_{n}^{j}\right)g_{n}^{j}}{\sum_{n=1}^{N}G\left(\mu_{n},g_{n}^{j}\right)}<g^{j} \end{array}$$

The mode of signal structure satisfies $\left\|p_{n}^{j}\right\|=\left\|p^{j}\right\|$. So, ${\hat{b}}^{j}={ŷ}^{j}\cdot {\hat{p}}^{j}+{ĝ}^{j}\leq b_{n}^{j}$. Image patches meet the requirements of image intensity reduction after structural decomposition. Since ${\hat{b}}^{j}\in J\left(x\right)$ follows ${\hat{b}}^{j}\leq b_{n}^{j}$, *J*(*x*) ≤ *I*(*x*). So, the fused image always meets the requirements of image intensity reduction.

## 4. Experimental Analysis

### 4.1. Experiment Preparations

Eighty three real-world foggy natural images with different sizes are used in the comparative experiments. These images can be downloaded from http://live.ece.utexas.edu/research/fog/fade_defade.html, http://github.com/agaldran/amef_dehazing, http://github.com/JiamingMai/Color-Attenuation-Prior-Dehazing or captured by ourselves. A synthetic foggy image dataset (RESIDE) with 100 scene images (Li et al., 2019) downloaded from http://sites.google.com/view/reside-dehaze-datasets. One hundred remote-sensing geographic images were collected from Google Earth by ourselves. Seventeen real-world tunnel images were collected by ourselves. Thirteen image defogging methods are used for comparison, which are AMEF (Galdran, 2018), CAP (Zhu et al., 2015), CO (He et al., 2016), DCP (He et al., 2009), DEFADE (Choi et al., 2015), GPR (Fan et al., 2016), MAMF (Cho et al., 2018), OTE (Ling et al., 2018), WCD (Chiang and Chen, 2012), DehazeNet (Cai et al., 2016), FFA-Net (Qin et al., 2020), a novel fast single image dehazing algorithm based on artificial multiexposure image fusion (MIF) (Zhu et al., 2021) and the proposed defogging method. All the experiments were programmed by MATLAB 2016b and run on a desktop with an Intel I9-7900X@3.30 GHz CPU and 16.00 GB RAM.

### 4.2. Subjective Visual Evaluation

**Figure 5:**
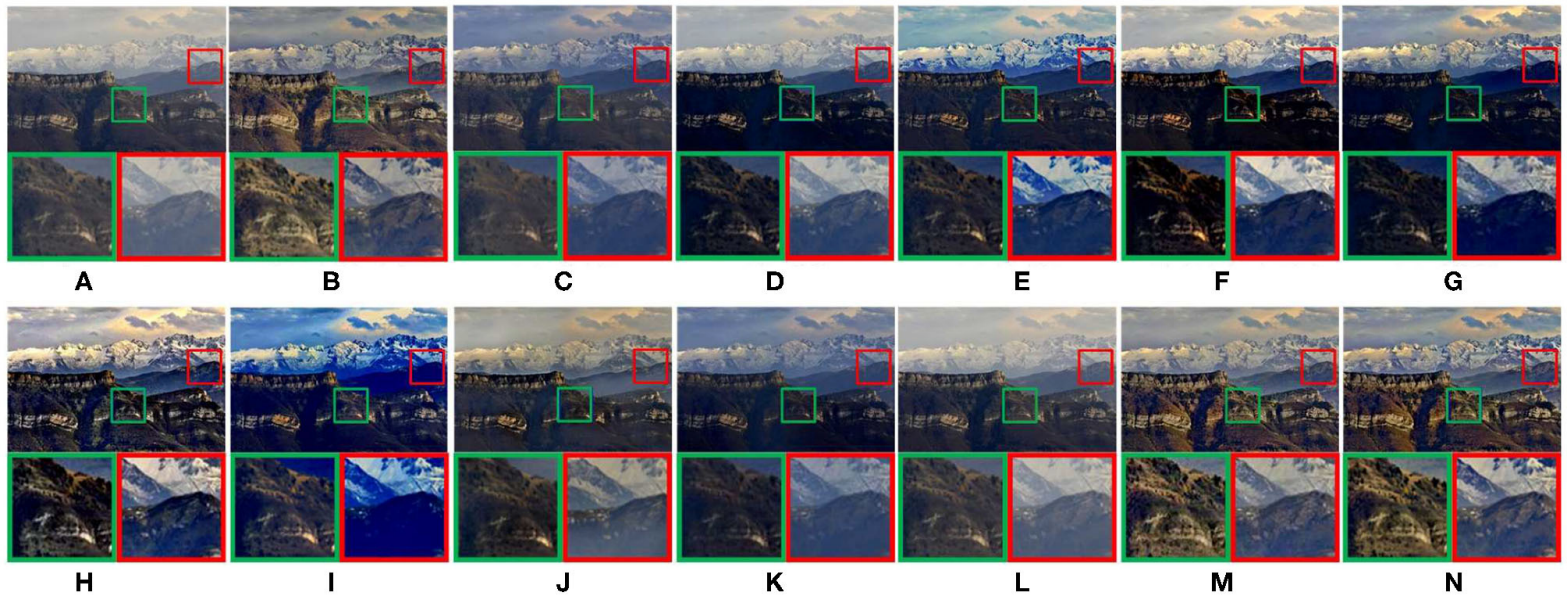
Real-world natural image. **(A)** Represents the original foggy image. **(B–N)** Represent foggy-free images processed by AMEF, CAP, CO, DCP, DEFADE, GPR, MAMF, OTE, WCD, DehazeNet, FFA-Net, MIF, and the proposed method.

**Figure 6:**
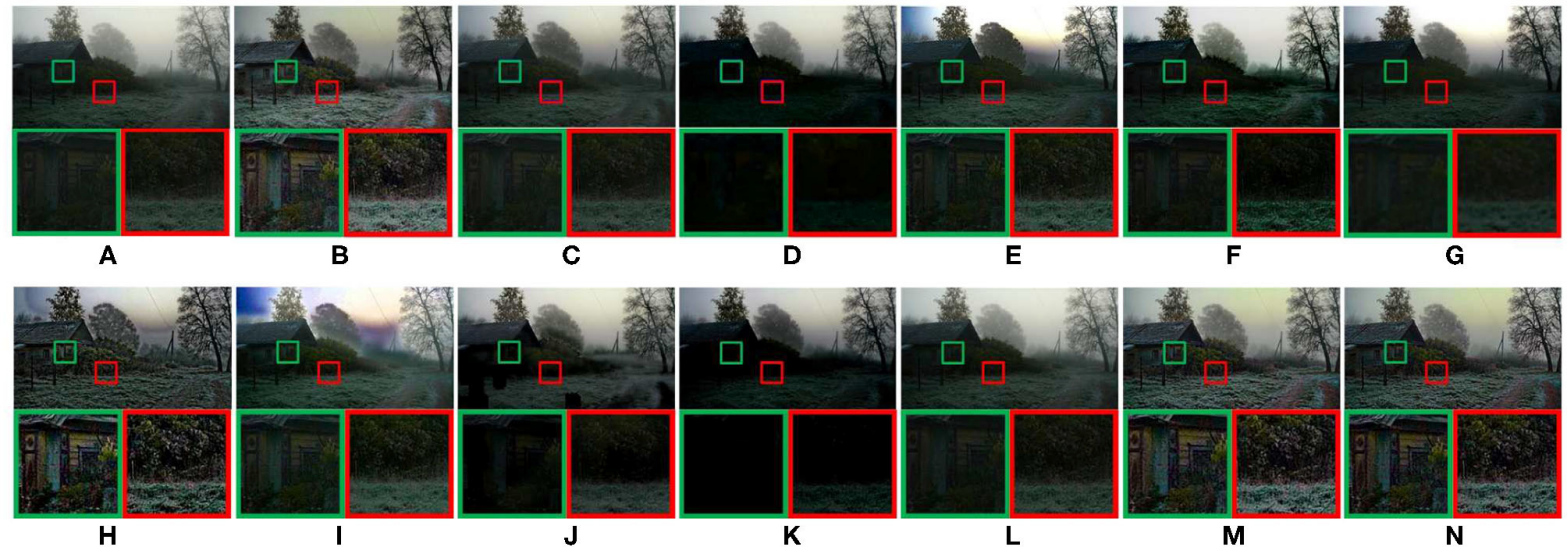
Real-world rural natural image. **(A)** Represents the original foggy image. **(B–N)** Represent foggy-free images processed by AMEF, CAP, CO, DCP, DEFADE, GPR, MAMF, OTE, WCD, DehazeNet, FFA-Net, MIF, and the proposed method.

**Figure 7:**
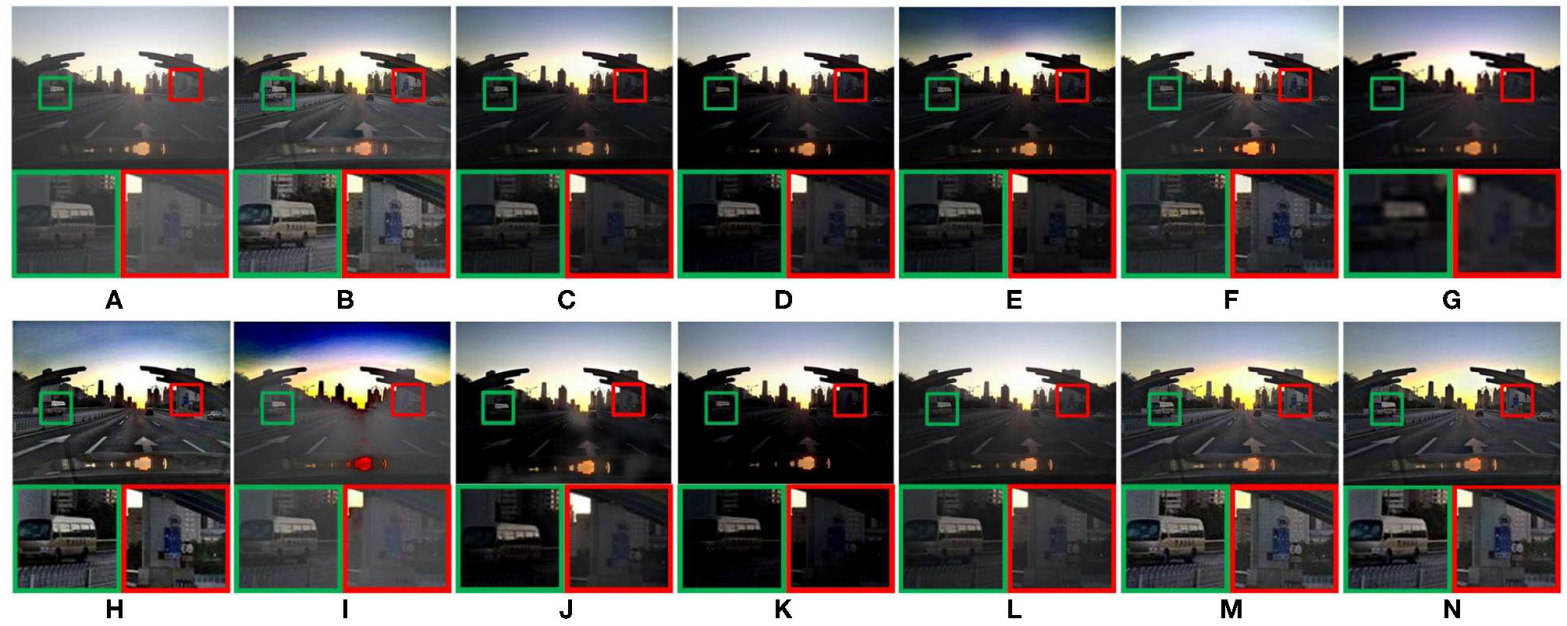
Synthetic driving image. **(A)** represents the original foggy image. **(B–N)** Represent foggy-free images processed by AMEF, CAP, CO, DCP, DEFADE, GPR, MAMF, OTE, WCD, DehazeNet, FFA-Net, MIF, and the proposed method.

**Figure 8:**
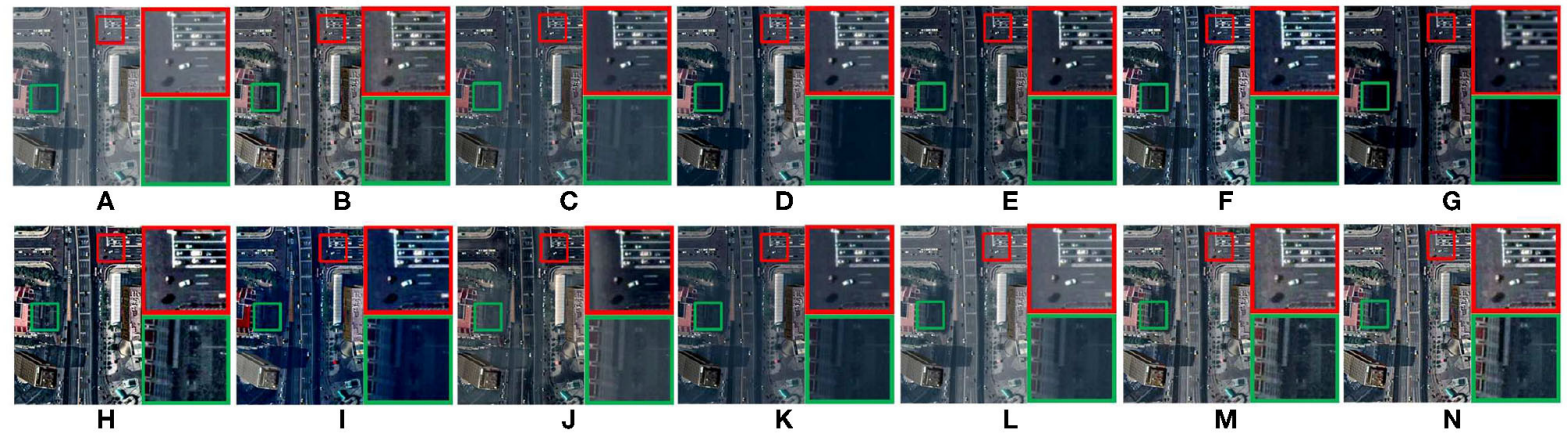
Remote-sensing geographic image. **(A)** Represents the original foggy image. **(B–N)** Represent foggy-free images processed by AMEF, CAP, CO, DCP, DEFADE, GPR, MAMF, OTE, WCD, DehazeNet, FFA-Net, MIF, and the proposed method.

**Figure 9:**
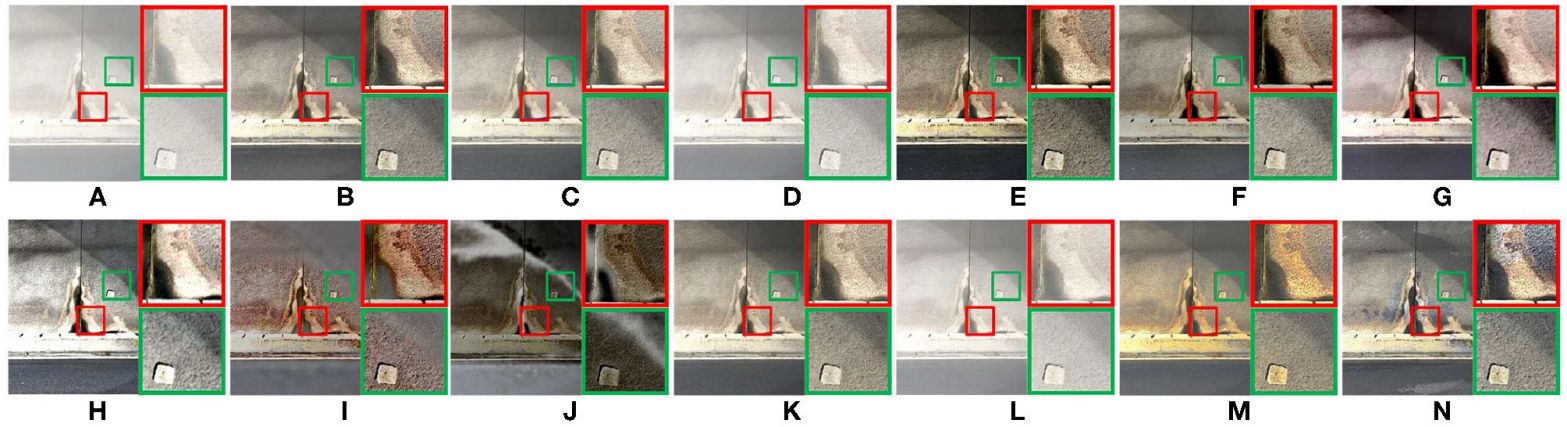
Tunnel image. **(A)** Represents the original foggy image. **(B–N)** Represent foggy-free images processed by AMEF, CAP, CO, DCP, DEFADE, GPR, MAMF, OTE, WCD, DehazeNet, FFA-Net, MIF, and the proposed method.

As shown in Figures 5–**9**. The results of five different scenes are selected to confirm that the proposed method has good defogging performance.

Figure 5 compares the defogging performance of thirteen methods on a real-world natural image. As shown in Figures 5C–K, the performance of CAP, CO, GPR, DehazeNet is poor. In the magnified areas, the details of the mountain are not visible. The hues shown in Figures 5E,I deviate. The global brightness of DEFADE and WCD as shown in Figures 5F,J respectively is low, and the fog shown in the magnified areas of Figure 5J is not completely removed. The brightness and saturation of Figure 5L are low. Although MAMF restores the high saturation of the source image, the contrast is sacrificed in the defogged image shown in Figure 5H, and the loss of structural and texture details can be seen from the magnified areas. As shown in Figures 5B–N, compared with other 10 methods, AMEF, MIF, and the proposed method achieve better defogging performance in local details and global brightness. The global saturation of the defogged image obtained by MIF or the proposed method is slightly better than the one obtained by AMEF.

Figure 6A is a real-world rural natural image. Due to the poor defogging performance of DCP and OTE, the color of sky is distorted, and the details shown in the magnified areas are lost, as shown in Figures 6E,I. In Figures 6D–L, the overall brightness of defogged images is too low, and the details shown in the magnified areas are lost. CAP and WCD have poor defogging performance. As shown in Figure 6C, there is no obvious change after defogging. The image saturation of Figure 6J is too low. As shown in Figures 6B, 7C–N, the image visibility is greatly improved, and the details shown in the magnified areas are clear. However, color distortion appears in the sky of Figure 6H. AMEF, MIF, and the proposed method have the best image defogging performance. The comparative results show that the overall brightness of the defogged image obtained by MIF or the proposed method is slightly better than the one obtained by AMEF.

Figure 7 compares the defogging performance of thirteen methods on a synthetic driving image. As shown in Figures 7E,I, the color of some areas in images is distorted, and the details shown in the magnified areas are lost. GPR have poor defogging performance, the clarity of the image decreased after defogging, as shown in Figure 7G. As shown in Figures 7C–K, the overall brightness of defogged images is too low, and the details shown in the magnified areas are lost. The sharpening degree of MAMF is too much, as shown in Figure 7H. In Figures 7F,L, some details information shown in the magnified areas are lost. As shown in Figures 7B–N, compared with other 10 methods, AMEF, MIF, and the proposed method have the best image defogging performance. The saturation of MIF and the proposed method is closer to the human eye observation habits than AMEF.

Figure 8 compares the defogging performance of 13 methods on a remote-sensing geographic image. As shown in Figures 8D–F, the details of the magnified areas are missing. The overall blurring degree of the defogged image obtained by GPR increases. The saturation of Figures 8H,I is too high, which leads to color distortion. The details of the magnified areas of Figure 8I are lost. As shown in Figure 8J, there is obvious contrast between light and dark light in the magnified areas. Figures 8B,K–N show good defogging performance, the overall brightness of the defogged images is good. However, the details shown in the magnified areas are lost, as shown in Figures 8K,L. After removing fog from the remote-sensing geographic image, it is helpful to recognize the objects shown in the remote-sensing geographic images and improve the recognition accuracy.

Figure 9 shows the defogged tunnel images obtained by 13 methods. The defogged image obtained by OTE has high saturation and color distortion, as shown in Figure 9I. In Figures 9C–L, obvious fog residue exists. The defogged image obtained by WCD has obvious distortion, as shown in Figure 9J. The overall brightness of Figure 9E is low. The overall brightness of Figures 9G,H is high, and the saturation is low. The saturation of Figure 9M is high. DEFADE, AMEF, DehazeNet, and the proposed method achieve good defogging performance. As shown in the magnified areas of Figure 9F, high saturation can reduce image contrast, and the texture details of tunnel wall are lost. After defogging tunnel images, the cracks on the inner wall of the tunnel and the pavement damages are well-observed.

### 4.3. Objective Evaluation

Structural similarity (SSIM) (Wang et al., 2004), peak-signal-to-noise ratio (PSNR) (Hore and Ziou, 2010), fog aware density evaluator (FADE) (Choi et al., 2015), and Entropy (Qing et al., 2016) are used as objective evaluation indexes. SSIM is used to measure the similarity between the defogged and reference images. The high SSIM value means the high similarity between the foggy and defogged images. PSNR is used to measure the distortion of defogging image compared with reference image. The high PSNR value means less distortion of defogging image. FADE is a no-reference evaluation index of image defogging performance. The image blurring degree is directly proportional to the value of FADE. Entropy reflects the average amount of information in the image. A large Entropy value means the large average amount of information is retained. Thirteen defogging methods are applied to 300 foggy images. Five defogged images are selected for illustration.

As shown in Table 1. According to the FADE and Entropy indexes of MAMF, MAMF can effectively reduce the fog density and retain the image information as much as possible. The Entropy of MIF and WCD is high, but FADE index of MIF and WCD reflects that MIF and WCD cannot effectively reduce the fog density. The FADE score is high, and the defogging performance is not effective enough. OTE and DEFADE can effectively reduce the fog density, but the Entropy of OTE and DEFADE rank low. In the defogging process, OTE and DEFADE lose some image information. The results of FADE and Entropy show that the proposed method can achieve good defogging performance.

**Table 1:** Evaluation of two objective indexes in the real-world natural image (Figure 5) defogging experiment.

	**AMEF**	**CAP**	**CO**	**DCP**	**DEFADE**	**GPR**	**MAMF**	**OTE**	**WCD**	**DehazeNet**	**FFA-Net**	**MIF**	**Proposed**
FADE	0.4177	0.7179	0.5151	0.3797	0.3121(4)	0.4086	0.2055(1)	0.2885(3)	0.5986	0.5366	0.8632	0.3414	0.2685(2)
Entropy	7.0971	6.7348	6.3071	7.0064	6.9570	6.7810	7.5853(1)	6.5808	7.0994(4)	6.9760	6.9084	7.3268(3)	7.3465(2)

In Table 2, FADE index of GPR and MAMF reflect that GPR and MAMF can effectively reduce the fog density, but Entropy index is low, some image information is lost in the defogging process. Entropy scores of FFA-Net and OTE are high, but their FADE indexes reflect that the defogging performance of FFA-Net and OTE are not good enough. MIF and the proposed method achieve a good ranking in FADE and Entropy indexes. MIF and the proposed method can effectively reduce the fog density and retain more image information.

**Table 2:** Evaluation of two objective indexes in the real-world rural natural image (Figure 6) defogging experiment.

	**AMEF**	**CAP**	**CO**	**DCP**	**DEFADE**	**GPR**	**MAMF**	**OTE**	**WCD**	**DehazeNet**	**FFA-Net**	**MIF**	**Proposed**
FADE	0.4169	0.7189	0.6898	0.4854	0.3776	0.3087(3)	0.1874(1)	0.5654	0.4919	0.3584	0.7282	0.3158(4)	0.2691(2)
Entropy	7.4687	7.2995	6.2526	6.3164	7.1563	7.3750	7.4412	7.5312(2)	7.1124	6.5062	7.5052(4)	7.5942(1)	7.5176(3)

As shown in Table 3, CAP, DEFADE, FFA-Net, and the proposed method have the highest four scores in SSIM index, which means that defogged result can effectively retain the structural information of the original image. However, PSNR index of CAP is low which means that there is more distortion in the defogging image. The PSNR of DehazeNet is high, but SSIM index of DehazeNet reflects that the structural information of the original image cannot be effectively preserved. DEFADE, FFA-Net and the proposed method achieve a good ranking in SSIM and PSNR indexes. DEFADE, FFA-Net, and the proposed method can effectively retain the structural information of the original image and reduce image distortion.

**Table 3:** Evaluation of two objective indexes in the synthetic driving image (Figure 7) defogging experiment.

	**AMEF**	**CAP**	**CO**	**DCP**	**DEFADE**	**GPR**	**MAMF**	**OTE**	**WCD**	**DehazeNet**	**FFA-Net**	**MIF**	**Proposed**
SSIM	0.8037	0.8904(3)	0.6737	0.7191	0.9273(2)	0.8221	0.7415	0.6733	0.5641	0.4868	0.9897(1)	0.8603	0.8645(4)
PSNR	29.198	25.983	26.114	24.650	33.323(3)	26.223	28.103	28.718	25.790	63.748(1)	37.461(2)	28.513	29.428(4)

As shown in Table 4, the Entropy index of AMEF and MIF reflects that AMEF and MIF can retain more image information in the process of defogging. But the FADE index ranking of AMEF and MIF is low, which proves that its defogging performance is poor. FADE index of OTE and DEFADE show that OTE and DEFADE can effectively reduce fog, but the score of Entropy is low. In the process of defogging, OTE and DEFADE lose some image information. MAMF and the proposed method achieve good results in FADE and Entropy. MAMF and the proposed method can ensure the high defogging performance and reduce the information loss during the defogging process.

**Table 4:** Evaluation of two objective indexes in the remote-sensing geographic image (Figure 8) defogging experiment.

	**AMEF**	**CAP**	**CO**	**DCP**	**DEFADE**	**GPR**	**MAMF**	**OTE**	**WCD**	**DehazeNet**	**FFA-Net**	**MIF**	**Proposed**
FADE	0.4201	0.6580	0.4681	0.3367	0.3028(4)	0.3852	0.1915(2)	0.2479(3)	0.4045	0.5049	0.7200	0.4105	0.1907(1)
Entropy	7.3273(3)	6.4009	6.6120	6.8067	7.2608	6.5937	7.4313(2)	6.5041	7.0765	6.7150	7.0420	7.3230(4)	7.5685(1)

According to FADE index in Table 5, DCP, OTE, WCD, and the proposed method can effectively reduce the fog density. However, the ranking of Entropy index of OTE and WCD show that more image information is lost in the defogging process. Entropy index of GPR and DehazeNet reflect that GPR and DehazeNet can retain most of image information in the defogging process, but the ranking of FADE index of GPR and DehazeNet is low. For DCP and the proposed method, their FADE and Entropy index rankings are high, which proves that they achieve good defogging performance and can effectively retain image information.

**Table 5:** Evaluation of two objective indexes in the tunnel image (Figure 9) defogging experiment.

	**AMEF**	**CAP**	**CO**	**DCP**	**DEFADE**	**GPR**	**MAMF**	**OTE**	**WCD**	**DehazeNet**	**FFA-Net**	**MIF**	**Proposed**
FADE	0.9207	1.1100	1.8038	0.5151(2)	0.6831	1.0563	1.0287	0.5799(4)	0.4712(1)	1.1700	1.5285	0.8143	0.5566(3)
Entropy	7.2576	7.3430	6.6227	7.5081(2)	7.1100	7.6589(1)	7.3797	6.8962	7.3096	7.4397(4)	6.9371	7.0303	7.4451(3)

The proposed method is more in line with human eye observation habits in color saturation, image brightness, and sharpness. The image details are effectively restored. In general, compared with the other 12 methods, the proposed method can achieve good defogging performance, reduce image distortion, and retain rich image information. For 300 foggy images, the average running time of AMEF, CAP, CO, DCP, DEFADE, GPR, MAMF, OTE, WCD, DehazeNet, FFA-Net, MIF, and the proposed methods were 2.8274, 3.1197, 6.1310, 3.4911, 85.7802, 433.5796, 3.9043, 38.7347, 7.3273, 7.6966, 302.5901, 1.8056, and 20.7910 s, respectively. Although the proposed method has good defogging performance and is widely used in various image scenes, the average processing time is relatively long owing to the high computational complexity.

## 5. Conclusion

The proposed method can effectively achieve fog removal without any a priori knowledge of the scene depth information. A single foggy image is first corrected by gamma correction, and then a set of underexposed images is obtained. Multi-exposure image set is composed of these underexposure images and the original foggy image. Next, the saturation of multi-exposure images is adjusted. The multi-exposure images are decomposed into the base and detail layers by a guided filter. The image details are enhanced by image patch decomposition. Low-level features such as mean intensity, signal strength, and signal structure are used to improve fusion quality. The best-quality areas are collected from each base-layer image patch for the fusion of image patches. The global exposure quality of the detail layer is optimized by using the global luminance components of each input image. The comparative experimental results confirm the effectiveness of the proposed method and its superiority over the state-of-the-art methods. The proposed method can be applied to natural images, synthetic images, remote-sensing geographic images, and tunnel images to improve image quality. This method includes image scale decomposition, exposure quality detection, base-layer image fusion, and detail-layer image fusion. These calculation processes can achieve effective image defogging, but also increase the computational complexity. In future, a simpler and more effective fusion strategy will be designed to reduce the calculation steps and the running time of image defogging, while maintaining defogging performance.

## Data Availability Statement

The original contributions presented in the study are included in the article/supplementary material, further inquiries can be directed to the corresponding author/s.

## Author Contributions

QL did data curation, formal analysis, conceptualization, formulated methodology, funding acquisition, software, visualization, and writing—original draft preparation. YL did formal analysis, investigation, devised the methodology, visualization, validation, and writing—review and editing. KL did investigation, resources, supervision, data curation, devised the methodology, and reviewed and edited the manuscript. WL did investigation, project administration, supervision, resources, and data curation. YC did supervision, resources, project administration, and funding acquisition. HD did formal analysis, conceptualization, resources, and data curation. XJ did supervision, data curation, investigation, project administration, and conceptualization. All authors contributed to this paper and approved the submitted version.

## Conflict of Interest

QL, KL, WL, HD, and XJ was employed by company China Merchants Chongqing Communications Technology Research & Design Institute Co., Ltd. The remaining authors declare that the research was conducted in the absence of any commercial or financial relationships that could be construed as a potential conflict of interest.
